# Vertebral Body Tethering in 49 Adolescent Patients after Peak Height Velocity for the Treatment of Idiopathic Scoliosis: 2–5 Year Follow-Up

**DOI:** 10.3390/jcm11113161

**Published:** 2022-06-02

**Authors:** James Meyers, Lily Eaker, Jessica Zhang, Theodor di Pauli von Treuheim, Baron Lonner

**Affiliations:** Department of Orthopaedic Surgery, Mount Sinai Hospital, New York, NY 10029, USA; james.meyers@icahn.mssm.edu (J.M.); lily.eaker@mountsinai.org (L.E.); jessica.zhang2@mountsinai.org (J.Z.); theodor.dipaulivontreuheim@icahn.mssm.edu (T.d.P.v.T.)

**Keywords:** vertebral body tethering, adolescent idiopathic scoliosis, non-fusion scoliosis correction

## Abstract

Vertebral Body Tethering (VBT) is a non-fusion surgical treatment for Adolescent Idiopathic Scoliosis (AIS) that elicits correction via growth modulation in skeletally immature patients. VBT after peak height velocity is controversial and is the subject of this study. A retrospective review of Risser 3–5 AIS patients treated with VBT, and min. 2-year FU was performed. Pre to post-op changes in clinical outcomes were compared using Student’s t-test or the Mann-Whitney test. A total of 49 patients met criteria, age 15.0 ± 1.9 years, FU 32.5 ± 9.1 months. For thoracic (T) major curvatures, T curvature improved from 51.1 ± 6.9° to 27.2° ± 8.1° (*p* < 0.01) and TL from 37.2° ± 10.7° to 19.2° ± 6.8° (*p* < 0.01). For thoracolumbar (TL) major curvatures, T improved from 37.2° ± 10.7° to 18.8° ± 9.4° (*p* < 0.01) and TL from 49.0° ± 6.4° to 20.1° ± 8.5° (*p* < 0.01). Major curve inclinometer measurements and SRS-22 domains, except activity, improved significantly (*p* ≤ 0.05). At the latest FU, one (2%) patient required fusion of the T curve and revision of the TL tether due to curve progression in the previously uninstrumented T curve and tether breakage (TB) in the TL. Twenty (41%) patients experienced TB. VBT in AIS patients with limited remaining skeletal growth resulted in satisfactory clinical outcomes at the latest FU.

## 1. Introduction

Vertebral body tethering (VBT) is a non-fusion surgical option for the treatment of adolescent idiopathic scoliosis (AIS). It was approved by the FDA via the Humanitarian Device Exemption (HDE) pathway and is currently indicated for use in skeletally immature patients for whom growth modulation occurs via the Heuter-Volkmann principle. There is an increasing body of literature supporting the use of VBT; however clinical outcomes have been less predictable than those following posterior spinal fusion (PSF), the current gold standard for AIS. This appears due, in part, to the challenge posed in determining ideal surgical timing. In a cohort of 17 young patients (16 with open triradiate cartilage and 1 closed) Newton et al. reported a low clinical success rate of 10/17 (59%; defined as residual major curve <35° and no indication for PSF at the last follow-up) with a 41% revision rate in all patients [1]. Alanay et al. studied 31 patients with variable skeletal maturity, including patients with Sanders 6 and 7, maturity and found more mechanical complications in the less skeletally mature (Sanders 2) [2].

Clinical success can be defined by improvement of radiographic parameters, pain reduction, improved body image and durability of outcomes. Patient and family preferences and goals must be taken into account when recommending a procedure, acknowledging attributes and drawbacks as well as gaps in knowledge of a particular procedure and approach [3,4]. Some families may have an aversion to the idea of a spinal fusion procedure for their child, either due to perceived or actual negative impacts, its permanency, or a desire to maintain flexibility of the spine and leave options available for future innovations in the arena of pediatric scoliosis correction. It is in this context that some patients have been offered VBT despite little or no remaining skeletal growth. This has been performed only after extensive discussion on the established indications for the procedure based on the basic science and clinical basis for the procedure, i.e., growth modulation in the skeletally immature patient as well as the regulatory approval by the FDA and other similar agencies around the world. The families were informed that little or no published literature is available to support this approach in the patient who has surpassed the stage of peak height velocity, that tether breakage is expected at some time point and that this will likely result in at least some loss of correction and the possibility of further surgery including fusion. The possibility of bone and soft tissue remodeling conferring some stability to curve correction even in the face of tether breakage was also discussed, but that possibility has not been proven or shown as of yet. The families were also made aware of the possibility of more durable tether cords being developed in the future that could significantly expand the construct half-life, making a revision with a tether a possibility. The families were also told that spinal fusion was the gold standard with a proven track record of good outcomes despite some drawbacks such as back pain, disc degeneration, and junctional deformities, as well as its non-physiologic nature [5,6,7]. Only after this extended discussion, was consent for the surgery obtained. 

Unfortunately, a paucity of research on clinical outcomes in VBT for patients who are nearing, or at, skeletal maturity means there is a need to further elucidate clinical indications, ideal surgical timing, and limits of the procedure [2,8,9,10,11,12]. To that end, we sought to study the clinical outcomes of VBT, performed by a single surgeon, applied to adolescents after peak height velocity.

## 2. Materials and Methods

### 2.1. Patient Selection

An IRB-approved retrospective chart review was performed for VBT cases performed between 2016 and 2019. Inclusion criteria included AIS patients undergoing VBT, a minimum 2-year follow-up, and Risser 3–5. The VBT procedure was performed with the Zimmer Dynesys implant. All families and patients were informed of the off-label use of the implant. The thoracic curve implantation was performed as described previously [13]. The thoracolumbar curve implantation differed from that in the thoracic spine in that the vertebral body screw placement was as posterior as possible for the lumbar curve in order to preserve lumbar lordosis. In some patients, a second cord was utilized in an anterior row of screws in order to preserve the longevity of the construct. In those cases, the posterior cord was tensioned first, and the anterior tether was tensioned secondly to approximately the same tension as the first tether. 

### 2.2. Demographic, Radiographic, and Clinical Outcomes

Clinical, radiographic, and SRS-22 outcomes were collected at the pre-operative visit and at latest follow-up. Hand radiographs for Sanders staging were not routinely available. The proximal humerus ossification system (PHOS) was used to assess skeletal maturity at baseline from standard scoliosis PA radiographs, in addition to Risser staging [14,15]. Coronal plane parameters including proximal thoracic (PTC), thoracic (MT), and thoracolumbar/lumbar (TL) curvature as well as sagittal parameters including T5-T12 kyphosis and T12-S1 lordosis were assessed. Additional sub-analysis was conducted on patients stratified by whether they had major curve instrumentation alone, or bilateral instrumentation. For those with thoracic major curves, 3D T5-T12 kyphosis was calculated as described by Pravesh et al. [16]. All complications were assessed. Tether breakage was assessed radiographically by the coronal angulation between adjacent screws increasing 5 degrees or more between first erect and latest follow-up [1,10,17]. Clinical success has been previously defined elsewhere as residual major curvature of either ≤35° or ≤30° and no indication for PSF at latest follow-up [1,10]. We defined clinical success as residual major curvature of ≤30° and no indication for PSF at latest follow-up. Finally, the Fulcrum Bending Correction Index (FBCI) was used to determine the amount of major curve correction accounting for curve flexibility [18].

### 2.3. Statistical Analysis

Descriptive statistics were calculated for all variables. Normality was assessed using a Kolmogorov-Smirnov test. Pre-operative data were compared to latest radiographic, clinical, and SRS-22 follow-up data. Student t-tests and non-parametric Mann-Whitney tests were used for comparisons.

## 3. Results

A total of 49 consecutive patients treated with VBT were included (Table 1). The mean age at time of surgery was 15.0 ± 1.9 years with mean follow-up of 32.5 ± 9.1 months. There were 12 (25%) Risser 3 patients, 33 (67%) Risser 4, and 4 (8%) Risser 5. There was 1 (2%) patient who was proximal humerus ossification system (PHOS) stage 3A, 21 (43%) who were 3B, 18 (37%) who were 4, and 9 (18%) who were 5. Lenke curve types were 23 (47%) Lenke 1, 1 (2%) Lenke 2, 5 (10%) Lenke 3, 19 (39%) Lenke 5, 1 (2%) Lenke 6. 15 (30%) patients had thoracic curves instrumented, 18 (37%) had thoracolumbar instrumented, and 16 (33%) had both.

### 3.1. Radiographic and Inclinometer Outcomes

Patients with major thoracic versus thoracolumbar curvatures were assessed separately (Table 2 and Table 3).

For those with thoracic major curvatures, the mean preoperative Cobb angle was 51.1 ± 6.9° corrected to 27.2 ± 8.1° at latest follow-up (48% correction; *p* < 0.01). Improvement was also seen in both the compensatory proximal thoracic curvature from 25.0 ± 8.2° to 16.5 ± 7.3° (34% correction, *p* < 0.01) and compensatory thoracolumbar curvature from 37.2 ± 10.7° to 19.2 ± 6.8° (48% correction, *p* < 0.01). There were significant improvements in 3D T5-T12 kyphosis restoration from 6.3 ± 10.8° to 22.5 ± 9.1° at latest follow-up (*p* < 0.01). Thoracic inclinometer measurements improved from 13.5 ± 3.8° to 7.9 ± 3.5° (*p* < 0.01) at latest follow-up and thoracolumbar inclinometer measurements improved from 8.1 ± 4.2 to 2.7 ± 1.8 (*p* < 0.01). The Fulcrum Bending Correction Index (FBCI) for thoracic major curves was 92.6 ± 37.7%. When thoracic curves were separated by magnitude at latest follow-up ≤ 30° (*n* = 15/24 (63%)) were compared to those that were >30° (*n* = 9/24 [38%]), pre-operative flexibility rate was 59.1% ± 18.8% versus 55.7% ± 18.1%, respectively. Furthermore, FBCI was 102.0 ± 39.1% in the clinically successful group compared to 77.0 ± 31.3% in the non-clinically successful group.

Patients with major thoracic instrumentation only (*n* = 15) were compared to those with bilateral instrumentation (*n* = 9). Compared to patients with bilateral instrumentation, patients with only their major thoracic curve instrumented saw similar improvement in curve magnitudes (52.2° ± 7.6° to 26.6° ± 5.4° and 50.5° ± 6.7° to 27.5° ± 9.5° respectively, *p* = 0.56 and *p* = 0.78) and similar rates of major curve correction (48.6 ± 10.6% and 45.7 ± 17.6% respectively, *p* = 0.65). Preoperatively, those with bilateral instrumentation had significantly larger compensatory thoracolumbar curves than their single-curve counterparts (46.1° ± 9.0° and 31.8° ± 7.8°, *p* < 0.01) and a subsequently larger percent correction (58.5% ± 11.8% vs. 35.0% ± 28.9%, *p* = 0.01). Thoracolumbar compensatory curve magnitude at latest follow-up was similar (19.2° ± 7.7° and 20.8° ± 9.8°, *p* = 0.68).

For those with thoracolumbar major curvatures, the mean preoperative Cobb angle was 49.0 ± 6.4°corrected to 20.1 ± 8.5° at latest follow-up (59% correction, *p* < 0.01). There was also significant improvement in the compensatory thoracic curvature from 37.2 ± 10.7° to 18.8 ± 9.4° (50% correction, *p* < 0.01). T5-T12 kyphosis did not significantly change, correcting from 23.3 ± 12.1 to 27.3 ± 16.4 (*p* = 0.34). Similarly, T12-S1 lumbar lordosis was maintained remaining at 55° between baseline and follow-up (*p* = 0.89). Thoracic inclinometer measurements were relatively stable (4.1 ± 4.2° to 2.4 ± 2.5°; *p* = 0.15) but thoracolumbar rotation decreased from 16.3 ± 4.4° to 4.6 ± 2.7° (*p* < 0.01). When thoracolumbar curves were separated by magnitude at latest follow-up ≤ 30° (*n* = 23/25 (92%)) compared to those that were > 30° (*n* = 2/25), the pre-operative flexibility rate was 78.8 ± 15.2% versus 72.0 ± 17.4%. Furthermore, FBCI was 76.4 ± 2.7% in the clinically successful group compared to 69.2 ± 9.1% in the non-clinically successful group.

Patients with major thoracolumbar instrumentation only (*n* = 18) were compared to patients with bilateral instrumentation (*n* = 7). Compared to patients with bilateral instrumentation, patients with only their major thoracolumbar curve instrumented saw similar pre-operative to post-operative curve magnitudes (52.4 ± 5.4° to 20.6 ± 7.6° and 47.7 ± 6.4° to 21.9 ± 8.1° respectively, *p* =0.10 and *p* = 0.76) and similar rates of correction in their main thoracolumbar curves (59.7 ± 16.3% and 53.8 ± 16.3% respectively, *p* = 0.43). Those with bilateral instrumentation had significantly larger compensatory thoracic curves than their single-curve counterparts preoperatively (46.1° ± 6.2° vs. 27.1 ± 5.7°, *p* < 0.01) but with similar percent correction (53.2 ± 20.6% vs. 36.5 ± 22.7%; *p* = 0.10) and magnitude of the final thoracic compensatory curve (21.1 ± 8.2° vs. 16.8 ± 6.2°; *p* = 0.17).

### 3.2. Clinical Success

There were 37/49 (76%) patients who were deemed clinically successful and had residual major curves ≤ 30°. Of those, 22/25 (88%) had thoracolumbar major curves while 15/24 (63%) had thoracic major curves. Sub-analysis was performed comparing clinically successful patients to those who were not. There was no difference between groups in age (*p* = 0.87) or Risser staging distribution (*p* = 0.06). Those in the clinically unsuccessful group had significantly larger preoperative major Cobb magnitude (54.9 ± 6.7 vs. 48.7 ± 6.0; *p* < 0.01) as well as less preoperative flexibility of the major curve although this did not reach statistical significance (58.7 ± 18.3 vs. 71.0 ± 19.1; *p* = 0.06).

### 3.3. Complications and Patient Reported Outcomes

There were two major complications in this cohort. One patient developed late onset superior mesenteric artery syndrome (SMAS) 1 year following her VBT procedure requiring Ladd’s derotation surgery. The second patient required revision to posterior fusion of the compensatory thoracic curvature 3.1 years after the index procedure due to curve progression in the uninstrumented thoracic curve and tether breakage in the thoracolumbar curve. The tether was revised with a second row of screws and double cord.

There were 20 (41%) patients who showed evidence of cord breakage. Of those 20 patients, three individuals showed evidence of cord breakage at two levels (Table 4).

When stratified by major curve, thoracolumbar curves with broken tethers demonstrated a greater decline in percent correction, from 63.6 ± 15.7% to 46.6 ± 12.6%, than thoracic curves, which declined from 48.9 ± 13.5% to 41.6% ± 18.6% (*p* = 0.01). While one patient with a broken tether was revised to PSF and counted amongst the treatment failure cohort, the other 19/20 (95%) patients with tether breakage still performed at the same rate as those with tethers that did not break; that is, of the broken tethers, 4/19 (21%) did not meet criteria for clinical success which was similar to the 7/29 (24%) of patients with intact tethers (χ^2^ = 0.06, *p* = 0.80).

Patients reported subjective outcomes with SRS-22 scores; however preoperative data were available for only a subset (24/49 [49%]) of participants. In those participants, there were significant improvements in all SRS domains except for activity scores (Table 5).

## 4. Discussion

The performance of VBT in patients after peak height velocity is controversial, and is the subject of this study. Our data provide preliminary support for its use in select skeletally mature patients. Of the 49 patients in this study, 37 (76%) remain clinically successful with major curvature less than 30 degrees at mean latest follow-up of 32.5 months (range 23–64). This includes 19 patients with broken tethers at the time of latest FU. For patients with both major thoracic (51.1 ± 6.9° to 27.2 ± 8.1°; 48% correction) and major thoracolumbar (49.0 ± 6.4° to 20.1 ± 8.5°; 59% correction) curves, there was a significant improvement in major Cobb magnitude (*p* < 0.01). Patient-reported outcomes improved with all SRS-22 domains except activity. When analyzing groups by success or failure, the age and Risser staging did not differ; however, the major Cobb angle in the unsuccessful group was about 55°, while it was closer to 49° in the successful group, which suggests that for slightly larger curves, extra care must be taken to assure sufficient tension is applied to the construct. Differences in curve flexibility were seen but did not reach statistical significance. The successful group had about 12% greater flexibility, and given the biological plausibility, one can imagine that with a larger sample size, flexibility may be a significant determinant of clinical success [19].

VBT is indicated for skeletally immature individuals with the potential for growth modulation based on health regulatory organization guidance. However, ideal candidates must not be too skeletally immature, as this has been associated with an increased incidence of overcorrection and reduced outcome predictability [9,11]. On the other hand, patients with little or no remaining growth do not have the potential for further correction beyond the index operation via Heuter Volkman-mediated asymmetric growth. Yet, in the limited studies inclusive of more skeletally mature patients, overcorrection rates were reduced while maintaining acceptable rates of clinical success in the more mature patient. Hegde et al. reported on 10 skeletally mature patients (Sanders ≥7) and found a 71% correction rate of the major Cobb with no incidence of re-operation or conversion to fusion at latest follow-up [20]. Alanay et al. assessed VBT outcomes stratified by Sanders skeletal maturity and found 55% correction of the major Cobb with no incidence of mechanical complications or overcorrection in the Sanders 6–7 cohort as compared to a 60% mechanical complication rate and 80% overcorrection rate in the Sanders 2 cohort in their study [2]. Hoernschemeyer et al. also reported on a case series inclusive of Risser 3 and 4 patients (*n* = 11/29 patients) and found a 74% clinical success rate (residual curve ≤30°) and a 7% conversion to fusion rate [10]. Comparatively, Newton et al. reported on a cohort of premenarchal, Risser 0, 94% open TRC cohort and had a significantly lower clinical success rate of 59% (residual curve < 35°) with one patient undergoing conversion to fusion and an additional three in whom it was indicated. Furthermore, four patients required reoperation for tether removal due to complete correction or overcorrection [1].

It has been previously reported that significant correction is achievable intraoperatively at the index operation via VBT [11,21]. In a study of the outcomes of VBT stratified by skeletal maturity, Alanay et al. found, at a mean follow-up of 20.1 months, that those in the Sanders 6–7 cohort experienced 55% intraoperative correction with no evidence of growth modulation and further curve correction after surgery although stable maintenance of the major curve was achieved [2]. However, their study was limited by the small sample size of that cohort (*n* = 7) and, as noted by the authors, the need for longer follow-up. Furthermore, they did not report on Sanders 8 patients. Unfortunately, we did not have hand X-rays of many of our patients, preventing an analysis by Sander stage. All our patients were Risser 3–5, with 75% being Risser 4 or 5 and 98% had PHOS stage of 3B-5. The PHOS system is more granular than Risser and spans the entire continuum of growth, as opposed to Risser staging. Our patients, thus, can be assumed to largely have surpassed their peak height velocity. Similarly, others have reported intraoperative correction rates ranging from 40% to 76% [1,2,11]. Given that the amount of correction achieved on the operating table is at the discretion of the surgeon, our results support the notion that adequate intraoperative correction for skeletally mature patients is possible, impacted by curve magnitude and flexibility as our analysis has shown, and the primary concerns are longitudinal outcomes and maintenance of correction if the tether breaks. At latest follow-up we found no differences in clinical success between those with tether breakage and those without (15/19 = 79% and 22/29 = 76% respectively, *p* = 0.80). At the senior author’s institution, all patients and their families were advised of the risks, benefits, and unknown outcomes associated with VBT for all patients including the mature patient. The following points are emphasized for all patients considering VBT: spinal arthrodesis is the standard of care, good and reliable intermediate outcomes are achieved, the long-term outcomes of VBT are unknown, the procedure has been predicated on remaining growth, the likelihood that tether breakage will occur and may result in significant loss of correction along with back pain, and the possible need for revision. Families often state they do not want a procedure that permanently alters their child’s spine, and prefer one that leaves options for future innovation. One such innovation is the development of stronger tethers that have the potential for prolonged durability. Furthermore, the controversial nature of performing VBT in skeletally mature individuals is also highlighted. Only after extensive discussion did the families proceed with the procedure. Until additional guidance is available from basic science and preclinical research, patients and providers will need to base their decisions on knowns and unknowns alike.

Whether or not holding the scoliotic spine straight for a prolonged period can result in sufficient spinal remodeling, to maintain correction even with tether breakage, is currently unknown, but is not without some evidence. Earlier studies in small mammals showed that remodeling of both immature and mature vertebrae is achievable through mechanical loading. For example, McBride et al. imposed a 30° scoliotic curve in rat tails and reported that the resultant vertebral wedging in immature rats was largely through asymmetric growth, exhibiting the Hueter-Volkmann law. They also reported that wedging was observed in mature rat vertebrae, and that this was largely due to diaphyseal remodeling, similar to Wolff’s Law [22,23]. Moreover, mature bone remodeling, in two directions, under compressive and tensile forces, has been demonstrated in the field of orthodontics [24,25]. Under compressive forces mature bone has been shown to resorb, while tensile forces lead to osteogenic bone build-up [26,27]. Certainly, further research is still needed to elucidate the potential magnitude of vertebral body remodeling in mature spines.

Ultimately, families are counseled in a shared-decision-making paradigm about the fact that the basic science case for performing this procedure and subsequent regulatory clinical approval are based on remaining growth, and that long term outcomes are lacking. All patients and families are encouraged to consider the gold standard fusion and, in appropriately indicated cases, posterior dynamic correction (ApiFix, TM, Yokneam Illit, Israel).

Limitations of the current study included the lack of Sanders staging as it is the most commonly reported skeletal maturity indicator spanning the period prior to the adolescent growth spurt through skeletal maturity. This was not routinely performed for our patients previously given it requires an additional hand radiograph that was not routinely collected to reduce radiation exposure. However, to address this, PHOS staging was provided for granular skeletal maturity assessment. Numbers were not sufficient to stratify outcomes based on Risser stage 3, 4, or 5. This will be the subject of future studies. Future study is also required to better elucidate predictors of clinical success in VBT for skeletally mature patients. Our sub-analysis was limited due to the sample size of those who were unsuccessful but best results were seen in smaller and more flexible curves.

## 5. Conclusions

While the VBT procedure is currently indicated for skeletally immature patients for whom growth modulation occurs via the Hueter-Volkmann principle, we provide preliminary data for its use in patients past their peak height velocity.

## Figures and Tables

**Table 1:** Demographics of cohort.

Cohort Demographics (*n* = 49)	
Gender (F)	36 (74%)
Age	15.0 ± 1.9
Major Cobb	50.1 ± 6.7
Minor Cobb	35.1 ± 10.6
Risser 3|4|5	12 (25%)|33 (67%)|4 (8%)
PHOS 3A|3B|4|5	1 (2%)|21 (43%)|18 (37%)|9 (18%)
Lenke 1|2|3|5|6	23 (47%)|1 (2%)|5 (10%)|19 (39%)|1 (2%)
Mean Follow-Up (months)	32.5 ± 9.1
Instrumented Curve	
Thoracic	15 (30%)
Thoracolumbar	18 (37%)
Both	16 (33%)
Cords Used	
Single	31 (63%)
Double	18 (37%)

**Table 2:** Radiographic parameters.

	Pre-Op	Latest Follow-Up	*p*-Value
All Patients (*n* = 49)			
PTC (°) (% Correction)	15.8 ± 11.1	12.4 ± 7.5 (21.5%)	0.08
MT (°) (% Correction)	41.6 ± 12.9	22.5 ± 8.7 (45.9%)	<0.01
TL (°) (% Correction)	43.2 ± 10.6	20.9 ± 8.3 (51.6%)	<0.01
T5-T12 Kyphosis (°)	22.2 ± 11.1	25.6 ± 13.4	0.19
T12-S1 Lordosis (°)	55.4 ± 12.5	56.0 ± 12.4	0.82
Thoracic Major Curves (*n* = 24)			
PTC (°) (% Correction)	25.0 ± 8.2	16.5 ± 7.3 (34%)	<0.01
MT (°) (% Correction)	51.1 ± 6.9	27.2 ± 8.1 (47.7%)	<0.01
TL (°) (% Correction)	37.2 ± 10.7	19.2 ± 6.8 (48.4%)	<0.01
3D T5-T12 Kyphosis (°)	6.3 ± 10.8	22.5 ± 9.1	<0.01
T12-S1 Lordosis (°)	55.8 ± 13.3	54.8 ± 10.0	0.85
Thoracolumbar Major Curves (*n* = 25)			
PTC (°) (% Correction)	7.7 ± 5.6	9.3 ± 6.0 (−20.8%)	0.35
MT (°) (% Correction)	37.2 ± 10.7	18.8 ± 9.4 (49.5%)	<0.01
TL (°) (% Correction)	49.0 ± 6.4	20.1 ± 8.5 (59.0%)	<0.01
T5-T12 Kyphosis (°)	23.3 ± 12.1	27.3 ± 16.4	0.34
T12-S1 Lordosis (°)	55.0 ± 12.0	55.5 ± 12.2	0.89

**Table 3:** Inclinometer measurements.

	Pre-Op	Latest Follow-Up	*p*-Value
All Patients (*n* = 49)			
Thoracic (°) (% Correction)	8.6 ± 6.2	5.1 ± 4.1 (40.7%)	0.01
Thoracolumbar (°) (% Correction)	12.4 ± 5.9	3.7 ± 2.5 (70.2%)	<0.01
Thoracic Major Curves (*n* = 24)			
Thoracic (°) (% Correction)	13.5 ± 3.8	7.9 ± 3.5 (41.5%)	<0.01
Thoracolumbar (°) (% Correction)	8.1 ± 4.2	2.7 ± 1.8 (66.7%)	<0.01
Thoracolumbar Major Curves (*n* = 25)			
Thoracic (°) (% Correction)	4.1 ± 4.2	2.4 ± 2.5 (41.5%)	0.15
Thoracolumbar (°) (% Correction)	16.3 ± 4.4	4.6 ± 2.7 (71.8%)	<0.01

**Table 4:** Comparison of patients with broken and intact tethers at latest follow-up.

	Broken Tether (*n* = 19)	Intact Tether (*n* = 29) *	*p*-Value
All Curve Types			
PTC (°) (% Correction)	10.4 ± 8.1 (21.7%)	12.8 ± 7.7 (31.7%)	0.31 0.15
MT (°) (% Correction)	21.3 ± 9.7 (42.5%)	23.4 ± 8.2 (47.6%)	0.41 0.36
TL (°) (% Correction)	23.9 ± 5.9 (47.5%)	19.2 ± 9.2 (54.0%)	0.05 0.29
T5-T12 Kyphosis (°)	22.4 ± 9.0	26.5 ± 14.1	0.36
T12-S1 Lordosis (°)	55.5 ± 12.6	55.8 ± 13.6	0.95
Curves ≤ 30°	15/19 = 79%	22/29 = 76%	0.80
Major Thoracic			
	Broken Tether (*n* = 7)	Intact Tether (*n* = 17)	*p*-Value
PTC (°) (% Correction)	17.4 ± 5.5 (26.1%)	16.1 ± 8.0 (37.2%)	0.70 0.17
MT (°) (% Correction)	30.0 ± 6.0 (41.6%)	26.0 ± 8.6 (48.9%)	0.28 0.29
TL (°) (% Correction)	22.9 ± 6.8 (48.2%)	19.1 ± 9.6 (44.6%)	0.36 0.92
T5-T12 Kyphosis (°)	26.4 ± 8.1	22.6 ± 10.1	0.38
T12-S1 Lordosis (°)	57.7 ± 8.3	55.9 ± 10.8	0.70
Major Thoracolumbar			
	Broken Tether (*n* = 8)	Intact Tether (*n* = 16)*	*p*-Value
PTC (°) (% Correction)	7.4 ± 6.4 (12.9%)	8.1 ± 4.1 (19.8%)	0.60 0.45
MT (°) (% Correction)	16.6 ± 8.2 (39.3%)	19.8 ± 6.2 (46.3%)	0.22 0.85
TL (°) (% Correction)	25.5 ± 5.2 (46.6%)	19.3 ± 9.1 (63.6%)	0.10 0.01
T5-T12 Kyphosis (°)	23.9 ± 17.1	32.3 ± 17.5	0.22
T12-S1 Lordosis (°)	57.4 ± 11.2	55.6 ± 17.3	0.99

* There was one revision that was not included in either broken or intact numbers as a final fusion was performed.

**Table 5:** SRS outcomes.

	Pre-Op	Latest Follow-Up	*p*-Value
All Patients (*n* = 24)			
Activity	4.2 ± 0.7	4.2 ± 0.4	0.5
Pain	3.8 ± 0.7	4.4 ± 0.6	0.02
Self-Image	3.4 ± 0.7	4.1 ± 0.5	<0.01
Mental	3.9 ± 0.7	4.2 ± 0.4	0.05
Satisfaction	3.3 ± 0.8	4.3 ± 0.6	<0.01
Mean	3.8 ± 0.5	4.2 ± 0.4	0.01

## Data Availability

Data will be maintained for 7 years post-publication of this study as required by local IRB regulations.
